# Associations Between Alzheimer’s Disease and Stroke: Clinical Studies of NA‐831 for AD and NA‐911 for Stroke

**DOI:** 10.1002/alz.095736

**Published:** 2025-01-09

**Authors:** Lloyd Tran, Zung Vu Tran, Fern Vu

**Affiliations:** ^1^ Biomed Industries, Inc., San Jose, CA USA

## Abstract

**Background:**

NA‐831 is a candidate for the treatment of Alzheimer’s Disease (AD). NA‐911 is an analog of NA‐831, serving as an IGF‐1 and GLP‐1 agonists. Animal studies of NA‐911 are evaluated for the treatment of by hypoxic‐ischemic injury, hemorrhagic stroke, and chronic neurodegenerative disorders

**Method:**

For NA‐831: A randomized clinical trial of NA‐831 was performed in 112 participants with mild and moderate AD, half received the drugs and half received placebo. The patients with MCI received 10 mg of NA‐831 or placebo orally per day. The patients with mild and moderate AD received 30 mg of NA‐831 or placebo orally per day. Subjects with MCI to meet the NIA‐AA core clinical criteria, CDR score of 0.5 and a Memory Box score of 0.5 or greater at Screening and Baseline. MMSE score ≥22. Subjects with mild & moderate AD to meet the NIA‐AA core clinical criteria. MMSE: 17‐21.

For NA‐911: We conducted animal studies of NA‐911 on adult male Sprague‐Dawley rats using the middle coronary artery occlusion (MCAO) method. This method is designed to mimic neurological and behavioral signs and symptoms of stroke in humans. Details of the animal study method will be described.

**Results:**

NA‐831 showed a significant improvement for patients with mild and moderate AD with the ADAS‐Cog‐13 score change of an average of 4.1 as compared to the placebo after 24 weeks of treatment (p = 0.001; ITT). CIBIC‐Plus showed 78% patients improved (p = 0.01; ITT). mNA‐831 was well‐tolerated at 30 mg/day.

In the animal study of NA‐911, the area of infarct in animals treated with vehicle was 43.4±7.4 mm^2^ (n = 13, Treatment with NA‐911 (3 mg/kg/h) significantly reduced the area of the infarct to 17.3 ±5.4 mm^2^ when compared to its vehicle treated group (n = 15, * P<0.05). It has been shown that progenitor cells residing in the SVZ are potentially able to replace the loss of nervous tissue after stroke.

**Conclusion:**

An association of Alzheimer’s disease and stroke has been suggested with clinical studies of NA‐831 and NA‐911. However, whether this association is causal requires further evaluation.

